# Development and validation of an updated PICC length prediction formula based on anteroposterior chest radiographs for the ultrasound-guided bedside placement

**DOI:** 10.1371/journal.pone.0294598

**Published:** 2023-11-21

**Authors:** Hyoung Nam Lee, Sangjoon Lee, Sung-Joon Park, Youngjong Cho, Hwan Hoon Chung

**Affiliations:** 1 Department of Radiology, Soonchunhyang University College of Medicine, Cheonan Hospital, Cheonan, Republic of Korea; 2 Vascular Center, The Eutteum Orthopedic Surgery Hospital, Paju, Republic of Korea; 3 Department of Radiology, Korea University College of Medicine, Korea University Ansan Hospital, Ansan, Republic of Korea; 4 Department of Radiology, University of Ulsan College of Medicine, Gangneung Asan Hospital, Gangneung, Republic of Korea; Stanford University School of Medicine, UNITED STATES

## Abstract

Bedside peripherally inserted central catheter (PICC) placement is sometimes required when the patient’s intrahospital transport is restricted, and the ideal catheter length prediction is needed. This study aimed to develop an updated formula that predicts the optimal length of a PICC based on anteroposterior chest radiographs (AP-CXRs). This retrospective study collected PICC procedure data as the training and validation sets in three hospitals, including cubital crease-puncture point distance (CP), the actual PICC length (aCL), and the approach side. Horizontal and vertical measurement variables were set on the AP-CXRs. Two dependent variables were ipsilateral upper arm length (AL) and ideal truncal catheter length (iTCL). Simple and multiple regression analyses were used for formula development, and it was applied to the test set to evaluate the length prediction performance. The study included 309 patients in the training and validation sets and 91 intensive care patients in the test set. The final derived formula was: (AL + iTCL = CP + estimated PICC length, cm) = 19.831 − 0.062 × (contralateral clavicle length, cm) + 0.255 × (2nd ribs horizontal distance, cm) + 0.720 × (humero-vertebral distance, cm) + 0.761 × (thoraco-carinal distance, cm) + 1.024 × (the vertical distance of two vertebral body units, cm). (If approaching from the left, add 2.843cm, and if female, subtract 0.821cm.) In the test set, there was no case of length prediction failure. Moreover, the catheter tip position was evaluated as optimal in 82 cases (90.1%). This study’s results suggest an updated formula to predict the ideal PICC length using only AP-CXRs for bedside placement.

## Introduction

Adequate and stable intravenous access is crucial for medical treatments. The peripherally inserted central catheter (PICC) is commonly used for long-term placement in critical care due to its low risk of complications [1, 2]. Various methods have been devised to predict the ideal catheter length for bedside PICC placement, including methods using bone and soft tissue landmarks [3–6], and those using the patient’s height [7–9].

Previously Park et al. reported the PICC length prediction formula using anteroposterior chest radiographs (AP-CXRs), which has the advantage of not requiring direct contact with the patient’s torso or information on the patient’s height [10]. However, their study had some limitations. In estimating the position of the catheter tip, not AP-CXR but fluoroscopy was used, so there is a mixed modality aspect. Moreover, because the length parameters were measured depending only on the bony landmark on AP-CXR, anatomical changes, such as tracheal deviation, were not reflected.

This study aims to propose an updated formula using AP-CXRs to predict appropriate PICC length in adult patients, taking into account factors such as vascular deviation, arm length, and different 0-points for various catheter manufacturers.

## Material and methods

This study was retrospective, and formal consent was not required. This study obtained the Institutional Review Board (IRB) approvals from all the participating institutions, and the need for informed consent was waived (IRB approval number of Institution A: 2023AS0098, B: 0749-220304-HR-056-01, C: SCHCA2023-05-026). The data were accessed for research purposes from April 2022 to June 2023 and the access period varied according to each hospital’s IRB approval date. For all data, all personally identifiable information other than gender and age was deleted and a separate serial number was assigned so that authors could not identify individuals during or after data collection.

### Study’s population: Training and validation sets

This study involved patients who underwent PICC by interventional radiologists from September 2021 to February 2022 in three hospitals. Only cases with proper post-procedural AP-CXRs that included the medial margin of the ipsilateral humerus, the lateral margin of both clavicles, or the inferior endplate of the twelfth thoracic (T12) vertebra were included. Patients with left-sided superior vena cava or severe scoliosis were excluded. Unlike the previous study by Park et al. [10], this study also included cases with unilateral lung volume loss, compression fracture of thoracic vertebrae, and elevated diaphragm due to ascites or a large mass.

### Procedure and data acquisition

Four experienced interventional radiologists performed the PICC procedures. The cubital crease was used as a reference point. To designate the cubital crease, the arm was abducted and the elbow flexed at 90 degrees to establish the crease. Afterward, the middle straight line among the skin folds was set and marked as the cubital crease.

Ultrasound was used to determine the vein to be accessed, with the basilic vein typically chosen, but the brachial vein selected if necessary. The shortest distance between the cubital crease and the puncture point (CP) was measured. Under local anesthesia, a micropuncture needle from the PICC set (5-Fr, Power PICC; BD Biosciences, Salt Lake City, UT, USA; / 5-Fr, Turbo-Ject PICC Set; Cook Medical Inc., Bloomington, IN, USA; / 6-Fr, Xcela PICC; Navilyst Medical Inc., Glens Falls, NY, USA; / 5-Fr, UNIS PICC; Genoss Co., Ltd., Suwon, South Korea) was used to puncture the vein under real-time ultrasound guidance. The actual PICC length and approach side for the punctured vessel were recorded. The catheter was inserted at the puncture site until the hub contacts the skin (so that no catheter remained outside the body). Notably, the PICC length inserted into the body was recorded as the actual catheter length (aCL) instead of the printed number, as the 0-point on the catheter may not match the starting point beyond the hub, depending on the manufacturer.

### Parameter measurements from the post-procedural AP-CXR

**Fig 1:**
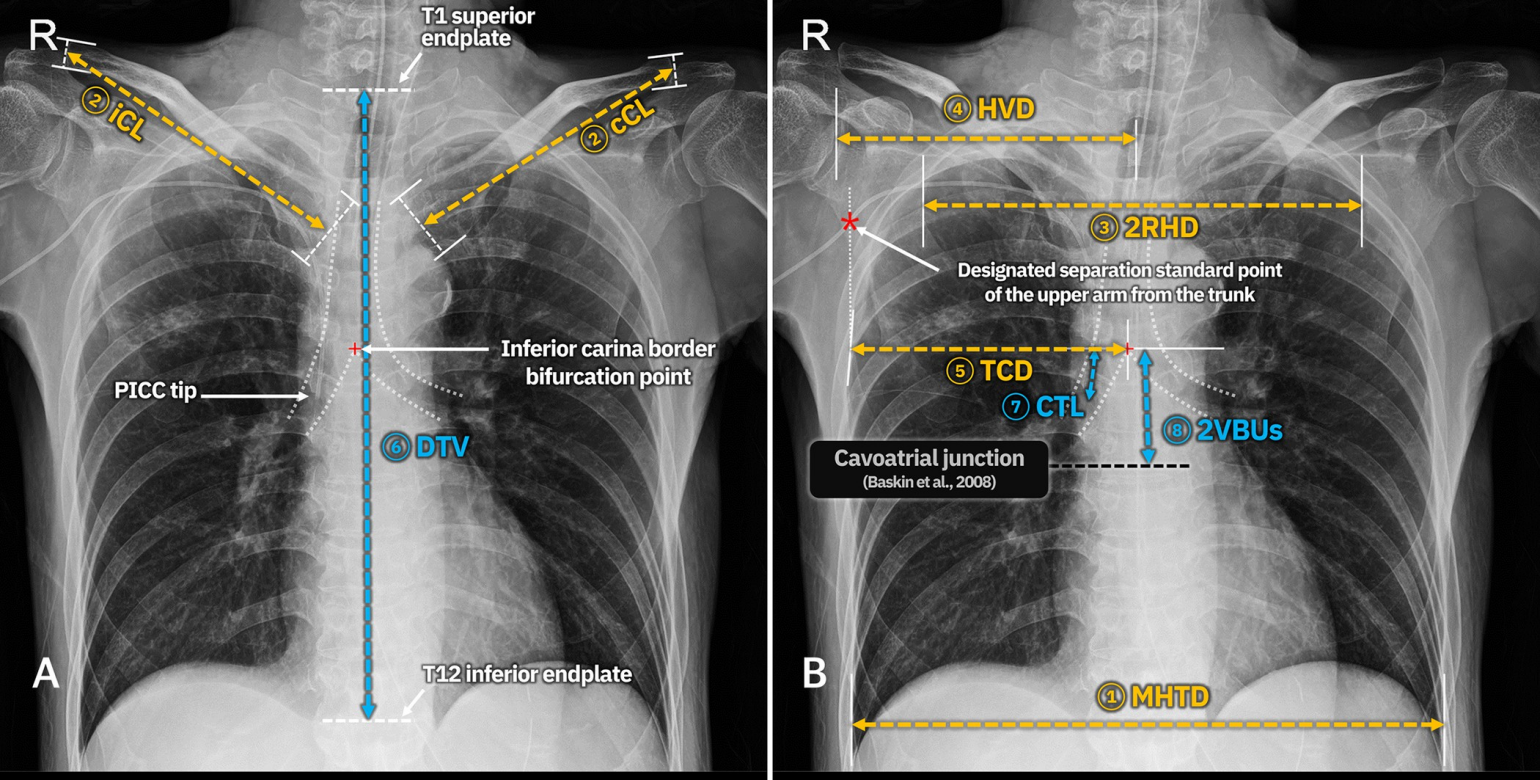
Radiologic parameters on anteroposterior chest radiographs. Horizontal elements are marked with yellow and vertical elements with blue. The parameters are as follows: MHTD, maximal horizontal thoracic diameter; iCL, ipsilateral clavicle length; cCL, contralateral clavicle length; 2RHD, second ribs horizontal distance; HVD, humero-vertebral distance; TCD, thoraco-carinal distance; DTV, distance of thoracic vertebrae; CTL, carina to catheter tip length; 2VBUs, vertical distance of two vertebral body units; T1, first thoracic vertebra; T12, twelfth thoracic vertebra; PICC, peripherally inserted central catheter.

Variables representing horizontal and vertical length elements were measured on post-procedural AP-CXRs using a picture archiving and communication system (Infinite G3; INFINITT Healthcare Co., Ltd., Seoul, South Korea). All AP-CXR images used in this study were taken by mobile digital radiographs at the bedside in the emergency room and intensive care unit. The set variables and their definitions were as shown in (Fig 1A and 1B), including the following: 1) maximal horizontal distance between the inner edges of the ribs (maximal horizontal thoracic diameter [MHTD]), 2) length between the midpoints of the clavicle’s proximal and distal ends (ipsilateral clavicle length [iCL]; contralateral clavicle length [cCL]), 3) maximal horizontal distance between the inner edges of the second ribs (second ribs horizontal distance [2RHD]), 4) shortest horizontal distance between ipsilateral humerus head medial border and vertebra midline (humero-vertebral distance [HVD]), 5) horizontal distance between the thoracic cavity lateral margin (parietal pleura, not visceral pleura; that is, an imaginary line connecting the inner edge of the rib) and carina bifurcation point at the level of the carina inferior border (thoraco-carinal distance [TCD]), 6) vertical distance from the superior endplate of the first thoracic (T1) vertebra to the inferior endplate of the T12 vertebra (distance of thoracic vertebrae [DTV]), 7) catheter length between the level of the inferior carina border to catheter tip (carina to catheter tip length [CTL]), and 8) two vertebral body units (2VBUs) below the inferior carina border. One VBU (vertebral body unit) was defined as the distance unit between the superior endplate of one vertebra to the superior endplate of the next, with the intervertebral disk included. The 2VBUs below the inferior carina border were designated as the cavoatrial junction (CAJ) according to a previous study by Baskin et al. [11]. If the catheter tip did not reach the designated CAJ, the length of the imaginary extension line of the catheter between the catheter tip and the 2VBUs below the carina inferior border was reflected as a negative CTL value.

**Fig 2:**
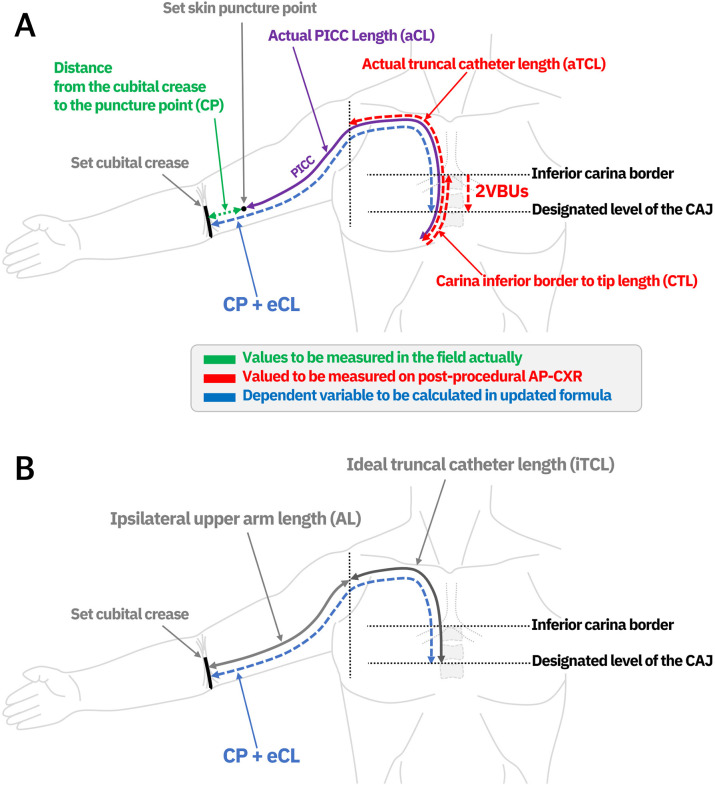
Schematic illustrations of parameters. (a) The distance from the cubital crease to the puncture point (CP) was measured in the field actually, and the actual catheter length (aCL) was also recorded. The actual truncal catheter length (aTCL), carina to catheter tip length (CTL), and vertical distance of two vertebral body units (2VBUs) were measured on the AP-CXRs. (b) The result of subtracting the CTL from the aTCL and adding the 2VBUs was defined as the ideal truncal catheter length (iTCL), to reach the cavoatrial junction (CAJ). The result of subtracting aTCL from CP + aCL was defined as the ipsilateral upper arm length (AL). eCL, estimated catheter length; PICC, peripherally inserted central catheter.

In this study, two dependent variables were set: ideal truncal catheter length (iTCL) and ipsilateral upper arm length (AL). The intersection point of the catheter and vertical line passing through the ipsilateral thoracic cavity lateral margin at the level of the carina inferior border on the AP-CXR was designated as the separation standard point of the upper arm from the trunk (Fig 1B). The catheter length from this point to the catheter tip was defined as the truncal catheter length (TCL). The measurement of the TCL on the post-procedural AP-CXR was defined as the actual TCL (aTCL). The result of subtracting the CTL from the aTCL and adding 2VBUs was defined as the iTCL, which was regarded as the ideal TCL to reach the CAJ. The result of subtracting the aTCL from CP + aCL was defined as the AL. The concept of the defined dependent variables is diagrammed in Fig 2.

### Statistical analyses

Statistical analyses were performed using SPSS (version 25.0; IBM Corporation, Armonk, NY, USA) [12], and *p*-values < 0.05 were considered statistically significant. Outliers were defined as values above or below 1.5 × IQR (the interquartile range) of the 1st to 3rd quartile for each variable and excluded before analysis. Simple regression analyses were performed between dependent and independent variables. Statistically significant variables and the approach side variable were applied to multiple regression analyses.

Data were divided into a training set for multiple regression analysis and a validation set to validate the formula. To prevent overfitting in a specific institution and to verify the reproducibility of the formula, the data for formula development were divided according to the institution unit. Three institutions participated in this study; two of them were separated as the training set, and multiple regression analysis was performed using data from these institutions. The data from the other institution was used for the validation set. Among them, the formula with the highest coefficient of determination (R^2^) in the training set and the lowest residual average in the validation set was selected. Multiple regression analyses were conducted using a stepwise method.

Formulas for dependent variables derived from the multiple regression test were combined into one formula and simplified. The sum of the AL and iTCL calculated through the formula was the same as calculating the sum of CP and estimated catheter length (eCL).

### Testing

**Fig 3:**
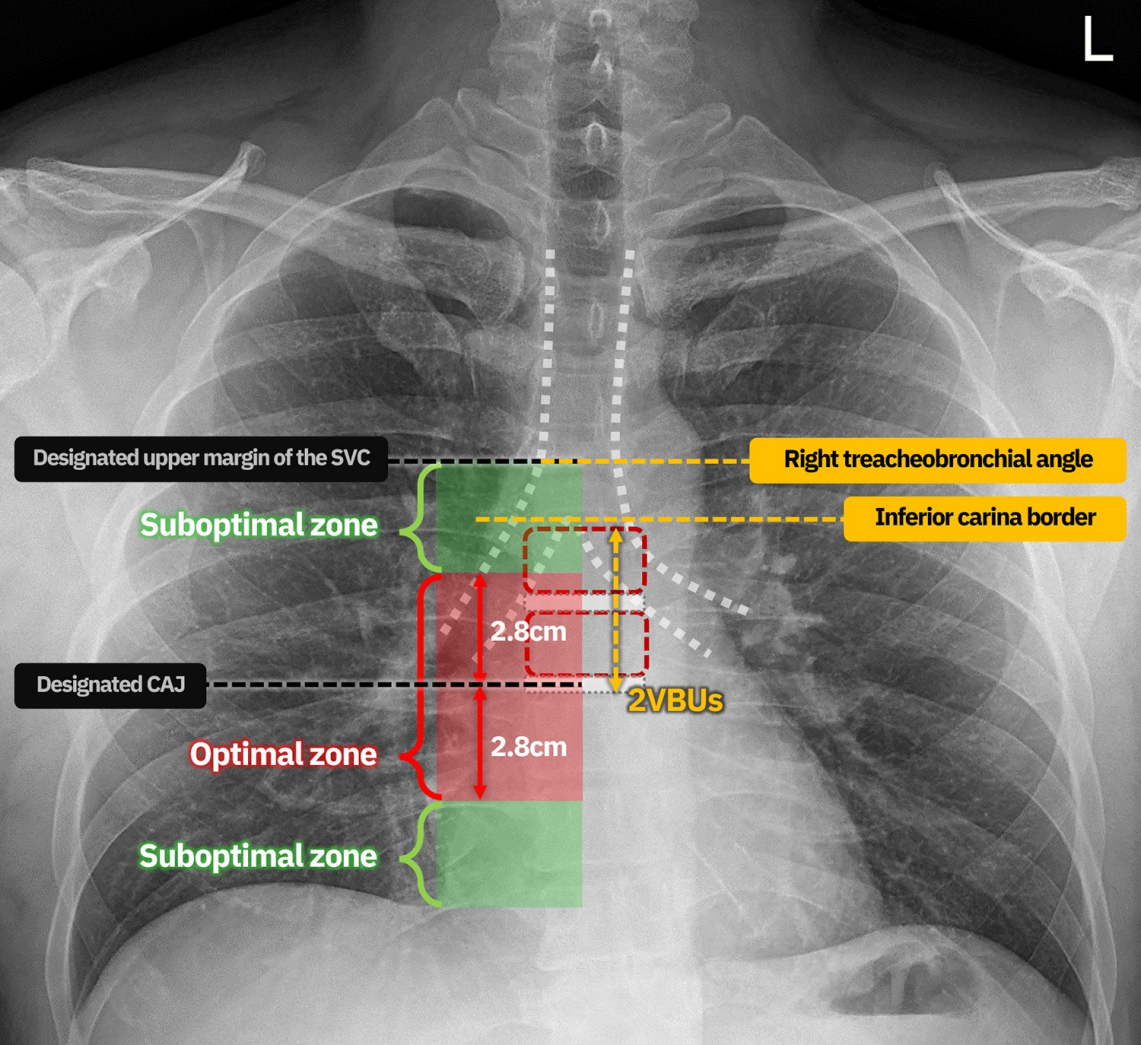
Illustration of the criterion for evaluating the success of length prediction. The results were classified as optimal or suboptimal. In the optimal position, the tip of the catheter was located in the range of approximately 2.8cm above and below the designated cavoatrial junction (CAJ) on the anteroposterior chest radiographs. In the suboptimal position, the catheter tip was positioned in the superior vena cava (SVC) zone (below the designated upper margin of the SVC) or right atrium. 2VBUs, vertical distance of two vertebral body units.

The derived model by multiple regression analysis was applied to the test set data to evaluate the success rate of PICC length prediction. The medical records of patients over 18 years of age who underwent ultrasound-guided bedside PICC placement without fluoroscopic guidance between February and August 2022 were reviewed using this formula. The inclusion criteria were the same as those used for the training set. The prediction success of catheter length was evaluated according to the estimated catheter tip position compared to the actual catheter tip position, comparing between eCL and aCL; the results were classified as optimal or suboptimal (Fig 3). This criterion was set to be the same as that of Cho et al.’s study [13], which validated the initial formula of Park et al. [10]. The upper margin of the superior vena cava (SVC) was designated at the right tracheobronchial angle on the AP-CXR following Aslamy et al.’s study [14]. In the optimal position, the tip of the catheter was located in the range of approximately 2.8cm above and below the designated CAJ on the AP-CXR. In the suboptimal position, the catheter tip was positioned in the SVC zone (below the designated upper margin of the SVC) or the right atrium (RA). If the catheter tip was located at another position, it was defined as a prediction failure.

## Results

### Patient characteristics

Patient characteristics and descriptive statistics for training, validation, and test sets are shown in Table 1. A total of 309 patients were included in the training and validation sets for formula development, while 91 patients were in the test set. The mean patient age was 68.50 ± 14.81 years, with 200 male patients (50.0%). The right-side approach was used in 359 patients (89.8%). PICC was inserted via the basilic vein in 217 patients (54.3%) and the brachial vein in 183 patients (45.7%).

**Table 1:** Patient characteristics and measured variables on the anteroposterior chest radiographs.

	Total (n = 400)	Formula development (n = 309)		Test (n = 91)
		Training set, n = 234 (58.5%)	Validation set, n = 75 (18.8%)	Test set, n = 91 (22.7%)
**Age (years, mean ± SD)**	**68.50 ± 14.81**	68.38 ± 15.22	74.48 ± 12.71	63.86 ± 13.72
**Sex (n)**				
Male	**200 (50.0%)**	114	32	54
Female	**200 (50.0%)**	120	43	37
**Side (n)**				
Right	**359 (89.8%)**	208	70	81
Left	**41 (10.2%)**	26	5	10
**Vessel (n)**				
Basilic vein	**217 (54.3%)**	127	32	58
Brachial vein	**183 (45.7%)**	107	43	33

SD: standard deviation

### Simple regression analyses for variable selection

The results from simple regression analyses between two dependent variables (AL and iTCL) and various independent variables (measured variables and patient characteristic variables) are listed in Table 2. There were statistically significant correlations between the AL and the following variables: sex, age, MHTD, iCL, cCL, 2RHD, HVD, TCD, DTV, and 2VBUs. There were statistically significant correlations between the iTCL and the following variables: sex, vessel, MHTD, iCL, cCL, 2RHD, HVD, TCD, DTV, and 2VBUs.

**Table 2:** Simple regression analyses for variable selection.

	Dependent variables							
	Ipsilateral upper arm length (AL)				Ideal truncal catheter length (iTCL)			
Independent variables	Regression coefficient	r	R^2^	*p*-value	Regression coefficient	r	R^2^	*p*-value
**Institution**	0.061	0.039	0.002	0.496	-1.189	0.091	0.008	0.112
**Sex (male: 0, female: 1)**	-0.738	0.215	0.046	**<0.001**	-2.309	0.508	0.258	**<0.001**
**Age**	-0.020	0.173	0.030	**0.002**	-0.007	0.044	0.002	0.441
**Vessel (basilic vein: 0, brachial vein: 1)**	-0.376	0.109	0.012	0.055	0.635	0.140	0.020	**0.014**
**MHTD (cm)**	0.265	0.333	0.111	**<0.001**	0.397	0.377	0.142	**<0.001**
**iCL (cm)**	0.560	0.420	0.176	**<0.001**	0.323	0.183	0.033	**0.001**
**cCL (cm)**	0.638	0.477	0.228	**<0.001**	0.302	0.171	0.029	**0.003**
**2RHD (cm)**	0.447	0.344	0.118	**<0.001**	0.632	0.367	0.135	**<0.001**
**HVD (cm)**	0.738	0.575	0.330	**<0.001**	0.517	0.304	0.092	**<0.001**
**TCD (cm)**	0.258	0.158	0.025	**0.005**	1.640	0.758	0.574	**<0.001**
**DTV (cm)**	0.155	0.305	0.093	**<0.001**	0.581	0.503	0.253	**<0.001**
**2VBUs (cm)**	1.355	0.302	0.091	**<0.001**	2.902	0.488	0.238	**<0.001**

MHTD: maximal horizontal thoracic diameter; iCL: ipsilateral clavicle length; cCL: contralateral clavicle length; 2RHD: second ribs horizontal distance; HVD: humero-vertebral distance; TCD: thoraco-carinal distance; DTV: distance of thoracic vertebrae; CTL: carina to catheter tip length; 2VBUs: two vertebral body units.

*p* < 0.05 were considered significant.

### Splitting training set and multiple regression analyses

Institution B’s cases (n = 75) were separated into the validation set. This showed the most significant R^2^ value (R^2^ of the AL formula: 0.474, R^2^ of the iTCL formula: 0.695) in a multiple regression analysis of the training set, and the lowest average of residual value of the validation set.

Table 3 shows the results from multiple regression analyses between dependent variables (AL and iTCL) and the aforementioned included variables.

**Table 3:** Result of multiple regression analyses using a stepwise method.

	Dependent variables							
	Ipsilateral upper arm length (AL) (R^2^ = 0.474, p<0.001, standard error = 1.31)				Ideal truncal catheter length (iTCL) (R^2^ = 0.683, p<0.001, standard error = 1.27)			
Variables	Regression coefficient	*p*-value	95% confidence interval		Regression coefficient	*p*-value	95% confidence interval	
**Constant**	**13.772**	<0.001	11.226	16.318	**6.059**	0.001	2.422	9.696
**Clinical data**								
Sex (Male: 0, Female: 1)					**− 0.821**	0.001	**−** 1.298	**−** 0.344
Approach side (R: 0, L: 1)	**0.956**	0.002	0.369	1.543	**1.887**	<0.001	1.324	2.450
**Horizontal elements**								
MHTD (cm)								
iCL (cm)								
cCL (cm)	**0.300**	0.001	0.128	0.472	**− 0.362**	<0.001	**−** 0.512	**−** 0.212
2RHD (cm)	**0.255**	0.005	0.077	0.434				
HVD (cm)	**0.720**	<0.001	0.558	0.882				
TCD (cm)	**− 0.502**	<0.001	**−** 0.737	**−** 0.267	**1.263**	<0.001	1.042	1.485
**Vertical elements**								
DTV (cm)								
2VBUs (cm)					**1.024**	<0.001	0.463	1.584

MHTD: maximal horizontal thoracic diameter; iCL: ipsilateral clavicle length; cCL: contralateral clavicle length; 2RHD: second ribs horizontal distance; HVD: humero-vertebral distance; TCD: thoraco-carinal distance; DTV: distance of thoracic vertebrae; 2VBUs: two vertebral body units.

Multiple regression analyses were constructed using a stepwise method.

*p* < 0.05 were considered significant.

Based on the multiple regression analysis results, the following formula to predict AL+iTCL (same as CP+eCL) was proposed:

(AL+iTCL=CP+eCL,cm)=19.831−0.062×(cCL,cm)+0.255×(2RHD,cm)+0.720×(HVD,cm)+0.761×(TCD,cm)+1.024×(2VBUs,cm)

**Fig 4:**
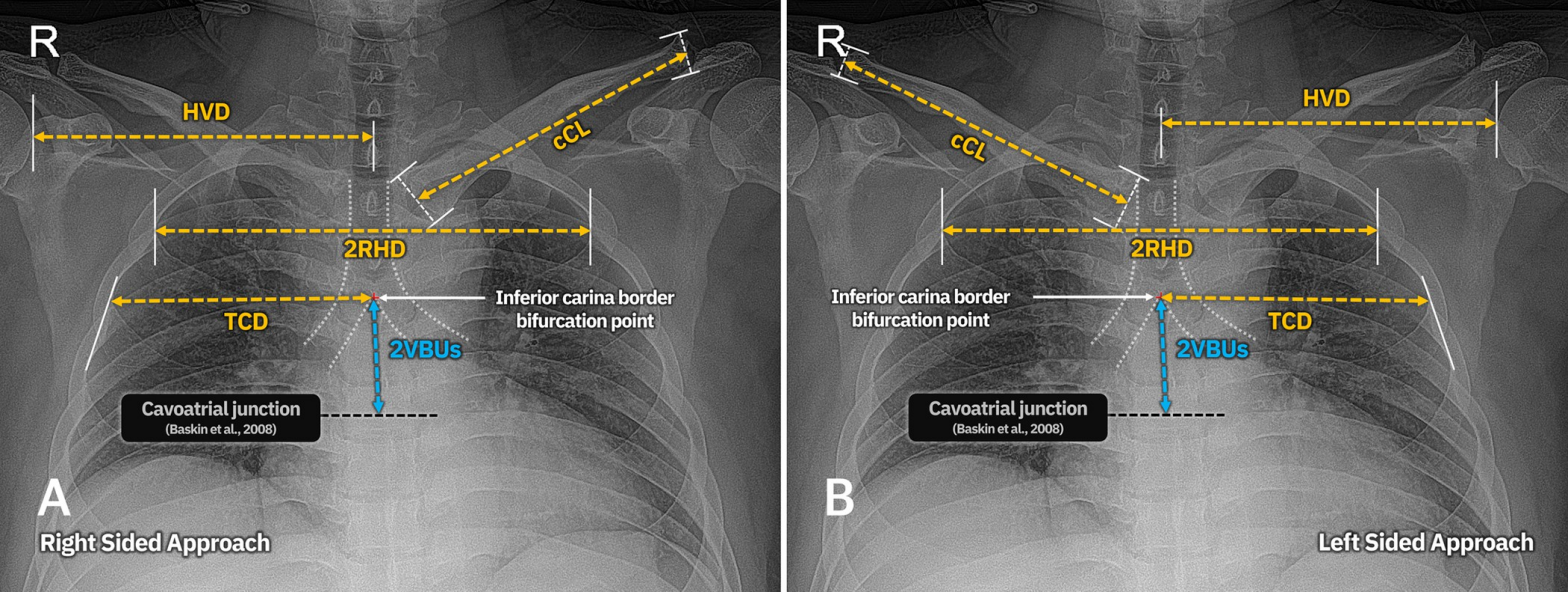
Variables measured on AP-CXR used in formula. (a) Measured variables when approaching from the right, (b) Measured variables when approaching from the left. cCL, contralateral clavicle length; 2RHD, second ribs horizontal distance; HVD, humero-vertebral distance; TCD, thoraco-carinal distance; 2VBUs, vertical distance of two vertebral body units.

If approaching from the left, add 2.843 cm; if female, subtract 0.821 cm (Fig 4).

To practically use the formula, the operator should designate the puncture point and measure the CP before the procedure. This value is subtracted from the calculated CP+eCL value which results in the eCL, and the catheter should be pre-cut to this length. At this time, an essential point not to forget is that if the 0-point on the catheter to be used is a certain distance away from the hub, the catheter should be cut at the point where the number subtracted by that distance printed.

To facilitate convenient calculations, we have created a standalone application that operates on Microsoft Windows and provided it via GitHub. This application was written for Python 3.8 or above and utilized the clipboard library which was freely available. The graphic user interface was constructed using PyQt5. The complete source code and graphic instances are available on GitHub at the following link: https://github.com/PSJCreX/BedsidePICC.

### PICC length prediction result in the test set

In the reviewed 91 cases of the test set with this formula, there were no prediction failures. Comparison of eCL to aCL suggested optimal catheter tip position prediction in 82 cases (90.1%) and suboptimal prediction in 9 cases (9.9%). Among suboptimal predictions, the catheter tip was predicted as located in the SVC in six cases and the RA in three cases, with no instances of catheters predicted as too deep below the heart contour.

## Discussion

There are many advantages to using PICCs in intensive care units, including a low complication risk during insertion, even in patients with altered coagulation [1, 6]. Compared with a central venous catheter via the internal jugular or subclavian vein, PICC insertion carries a low risk of pleuropulmonary damage or a local hemorrhage or hematoma [15], so this technique is performed worldwide. The placement of a PICC at the bedside has become increasingly common in various clinical settings [6, 13, 16–18]. However, determining the length of the catheter is one of the most challenging aspects of a successful bedside PICC procedure.

This study introduced an updated new formula for predicting PICC length using AP-CXRs, the most common chest radiograph in clinical settings. This formula achieved an optimal length prediction rate of 90.1%, which is more accurate than that using previous Park et al.’s formula of 70.2% validated by Cho et al. [10, 13]. Although the appropriate catheter tip position is still debated, the CAJ area is considered the most ideal on the premise that it is not wedging to the RA wall [11, 19–22]. Inserting the catheter too deep may cause arrhythmia or increase the risk of cardiac tamponade due to cardiac perforation [23–25].

There have been several methods that have attempted to predict length in bedside PICC placement without fluoroscopy. The first study using chest radiograph measurements to predict PICC length was reported by Ramamurthi et al. in child patients [26], and the first study using AP-CXR measurement elements in adult patients was reported by Park et al. [10]. The previous formula by Park et al. required improvements. Firstly, it mixed modalities, using fluoroscopy instead of AP-CXRs to determine PICC tip position. In contrast, this study’s formula relied solely on AP-CXRs. Secondly, the previous formula didn’t account for variations in horizontal length due to lung volume loss or mass. The previous formula used the MHTD and iCL as horizontal elements, which did not reflect trachea or vessel deviation because they were simply bony structures. This study added TCD as a variable to address horizontal anatomic changes. Thirdly, the prior formula didn’t consider the patient’s arm length, which was addressed in this study. In this study, arm length was set as a separate dependent variable to be predicted, and multiple regression analysis results showed the R^2^ of 0.474. Lastly, the distance from the hub to the 0-point, which varies between manufacturers, was not considered in the previous formula, but it was included in this study.

Other methods have been reported to determine the tip position of the central venous catheter. For example, it has been reported that electromagnetic positioning systems such as the Sherlock 3CG^TM^ Tip Confirmation System (BD Biosciences, Salt Lake City, UT, USA) or the Certofix/Alphacard (B.Braun Medical, Boulogne Cedex, France), can also be used. However, cost-effectiveness or distribution issues in several countries may make these systems unsuitable for general use [27, 28]. Cho et al. [29] describe a method of integrating portable digital radiography with bedside placement of PICCs. However, this may result in unnecessary radiation exposure to nearby patients and medical staff in environments without individual rooms. This study’s method using AP-CXRs has no additional costs. Although the bedside PICC placement is performed using only ultrasound, it can be inserted at a relatively accurate length using this study’s formula.

AP-CXR devices cannot maintain a constant source-image distance, which may affect magnification and introduce some measurement errors. However, AP-CXRs are commonly used in intensive care units and emergency rooms, so they were chosen as the primary modality for this study to reflect real-world clinical settings requiring bedside PICC placement. During taking an AP-CXR, the X-ray tube is usually raised at the maximum height of the machine to cover the entire torso of the patient in an image detector field. Considering the bed height, the actual source-image distance of the AP-CXR is estimated to be about 100~110 cm in the supine position, so it will not vary more than concerned.

This study has several limitations. First, this study was a retrospective design based on existing medical records. Second, since this study was aimed at Asians, it cannot be regarded as a formula representing all races. Third, using an AP-CXR to reflect actual clinical situations inevitably involves measurement errors. Moreover, since most patients had poor coordination or reduced consciousness, the AP-CXRs were taken without breathing control, which may have served as a limitation. Fourth, this formula can be applied after the cubital crease set; therefore, this formula will be difficult to apply in patients with difficult designation for the cubital crease due to redundant skin, burns, underweight conditions, or severe edema.

## Conclusion

In conclusion, this study suggests an updated formula to predict the ideal PICC length using only an AP-CXR for bedside placement. Based on the test results, the updated formula presented in this study will provide better PICC length prediction results for bedside procedures than the previous formula. Patients needing a PICC but that have restricted intra-hospital transport with hemodynamic instability or concerns about infection-related contamination, may benefit from this formula.

## Supporting information

S1 Data The data of training and validation groups. (XLSX)

## References

[1] Chopra V, Flanders SA, Saint S, Woller SC, O’Grady NP, Safdar N, et al. The Michigan Appropriateness Guide for Intravenous Catheters (MAGIC): Results From a Multispecialty Panel Using the RAND/UCLA Appropriateness Method. Ann Intern Med. 2015 Sep 15;163(6 Suppl):S1–40. doi: 10.7326/M15-0744 26369828

[2] Wilson TJ, Brown DL, Meurer WJ, Stetler WR, Wilkinson DA, Fletcher JJ. Risk factors associated with peripherally inserted central venous catheter-related large vein thrombosis in neurological intensive care patients. Intensive Care Med. 2012 Feb;38(2):272–8. doi: 10.1007/s00134-011-2418-7 22113818

[3] Kang SS, Shin YS, Lee SY, Kim H. Simplified equation for determining proper depth of peripherally inserted central catheter in relation to anatomical landmarks. Korean J Anesthesiol. 2018 Aug;71(4):300–4. doi: 10.4097/kja.d.18.27185 29684986 PMC6078871

[4] Kim MC, Kim KS, Choi YK, Kim DS, Kwon MI, Sung JK, et al. An estimation of right-and left-sided central venous catheter insertion depth using measurement of surface landmarks along the course of central veins. Anesth Analg. 2011 Jun;112(6):1371–4. doi: 10.1213/ANE.0b013e31820902bf 21233490

[5] Joshi S, Kulkarni A, Bhargava AK. Evaluation of length of central venous catheter inserted via cubital route in Indian patients. Indian J Crit Care Med Peer-Rev Off Publ Indian Soc Crit Care Med. 2010;14(4):180–4. doi: 10.4103/0972-5229.76081 21572748 PMC3085218

[6] Lim J, Chung CR, Ryu JA, Gil E. Bedside Ultrasound-Guided Peripherally Inserted Central Catheter Placement by Critical Care Fellows in Critically Ill Patients: A Feasibility and Safety Study. J Acute Care Surg. 2021 Mar 31;11(1):30–5.

[7] Jeon EY, Koh SH, Lee IJ, Ha H il, Park BJ. Useful Equation for Proper Estimate of Left Side Peripherally Inserted Central Venous Catheter Length in Relation to the Height. J Vasc Access. 2015 Jan 1;16(1):42–6. doi: 10.5301/jva.5000309 25362985

[8] Cho HH, Jeon EY, Lee HJ, Lee H, Koh SH, Choi SY, et al. A New Formula to Estimate the Length of Right Upper Extremity Vein from Elbow Crease to Carina Calculated by Peripherally Inserted Central Catheter Insertion through Right Basilic Vein Puncture. J Korean Soc Radiol. 2013 Sep 12;66(3):229–33.

[9] Lum P. A New Formula-Based Measurement Guide for Optimal Positioning of Central Venous Catheters. J Assoc Vasc Access. 2004 Jan 1;9(2):80–5.

[10] Park SJ, Chung HH, Lee SH, Kim JE, Kim C, Lee SM. New formulas to predict the length of a peripherally inserted central catheter based on anteroposterior chest radiographs. J Vasc Access. 2021 Mar 22;11297298211001148. doi: 10.1177/11297298211001147 33752491

[11] Baskin KM, Jimenez RM, Cahill AM, Jawad AF, Towbin RB. Cavoatrial junction and central venous anatomy: implications for central venous access tip position. J Vasc Interv Radiol JVIR. 2008 Mar;19(3):359–65. doi: 10.1016/j.jvir.2007.09.005 18295694

[12] IBM Corp. IBM SPSS Statistics for Windows [Internet]. Armonk, NY: IBM Corp; 2017. Available from: https://hadoop.apache.org

[13] Cho Y, Lee S, Park SJ, Lee HN, Chung HH. Validation of the PICC length prediction formula based on anteroposterior chest radiographs for bedside ultrasound-guided placement. PLOS ONE. 2022 11;17(11):e0277526. doi: 10.1371/journal.pone.0277526 36367880 PMC9651554

[14] Aslamy Z, Dewald CL, Heffner JE. MRI of central venous anatomy: implications for central venous catheter insertion. Chest. 1998 Sep;114(3):820–6. doi: 10.1378/chest.114.3.820 9743173

[15] Pittiruti M, Hamilton H, Biffi R, MacFie J, Pertkiewicz M. ESPEN Guidelines on Parenteral Nutrition: Central Venous Catheters (access, care, diagnosis and therapy of complications). Clin Nutr. 2009 Aug 1;28(4):365–77. doi: 10.1016/j.clnu.2009.03.015 19464090

[16] Kim YO, Chung CR, Gil E, Park CM, Suh GY, Ryu JA. Safety and feasibility of ultrasound-guided placement of peripherally inserted central catheter performed by neurointensivist in neurosurgery intensive care unit. PloS One. 2019;14(5):e0217641. doi: 10.1371/journal.pone.0217641 31150465 PMC6544252

[17] Kwon S, Son SM, Lee SH, Kim JH, Kim H, Kim JY, et al. Outcomes of bedside peripherally inserted central catheter placement: a retrospective study at a single institution. Acute Crit Care. 2020 Feb;35(1):31–7. doi: 10.4266/acc.2019.00731 32131579 PMC7056959

[18] Lee Y, Ryu JA, Kim YO, Gil E, Song YM. Safety and feasibility of ultrasound-guided insertion of peripherally inserted central catheter performed by an intensive care trainee. J Neurocritical Care. 2020 Mar 30;13(1):41–8.

[19] Vesely TM. Central venous catheter tip position: a continuing controversy. J Vasc Interv Radiol JVIR. 2003 May;14(5):527–34. doi: 10.1097/01.rvi.0000071097.76348.72 12761305

[20] Scott WL. Central venous catheters. An overview of Food and Drug Administration activities. Surg Oncol Clin N Am. 1995 Jul;4(3):377–93. 7552783

[21] Networks NA of VA. NAVAN. Position Statement. J Vasc Access Devices. 1998;3:8–10.

[22] Infusion Nurses Society. Infusion Nursing Standards of Practice. J Infus Nurs Off Publ Infus Nurses Soc. 2006 Feb;29(1 Suppl):S1–92. doi: 10.1097/00129804-200601001-00001 16429002

[23] Chabanier A, Dany F, Brutus P, Vergnoux H. Iatrogenic cardiac tamponade after central venous catheter. Clin Cardiol. 1988;11(2):91–9. doi: 10.1002/clc.4960110207 3345609

[24] Cevik M, Erek E. Hickman catheter-induced cardiac tamponade-related cardiac perforation management by mediastinotomy in children and a review of the literature. Trauma Case Rep. 2021;100436. doi: 10.1016/j.tcr.2021.100436 33665325 PMC7907533

[25] Amerasekera SSH, Jones CM, Patel R, Cleasby MJ. Imaging of the complications of peripherally inserted central venous catheters. Clin Radiol. 2009 Aug;64(8):832–40. doi: 10.1016/j.crad.2009.02.021 19589422

[26] Ramamurthi A, Chick JFB, Srinivasa RN, Hage AN, Grove JJ, Gemmete JJ, et al. Chest Radiograph Measurement Technique Facilitates Accurate Bedside Peripherally Inserted Central Catheter Placement in Children. Cardiovasc Intervent Radiol. 2018 Mar;41(3):443–8. doi: 10.1007/s00270-017-1857-0 29238870

[27] Yamagishi T, Ashida H, Igarashi T, Matsui Y, Nozawa Y, Higuchi T, et al. Clinical impact of the Sherlock 3CG® Tip Confirmation System for peripherally inserted central catheters. J Int Med Res. 2018 Dec;46(12):5176–82. 30178687 10.1177/0300060518793802 PMC6300932

[28] Bidgood C. Improving the patient experience with real-time PICC placement confirmation. Br J Nurs Mark Allen Publ. 2016 Jun 26;25(10):539–43. doi: 10.12968/bjon.2016.25.10.539 27231736

[29] Cho SB, Baek HJ, Park SE, Choi HC, Lee SM, Bae K, et al. Clinical feasibility and effectiveness of bedside peripherally inserted central catheter using portable digital radiography for patients in an intensive care unit. Medicine (Baltimore). 2019 Jun 28;98(26):e16197. 31261562 10.1097/MD.0000000000016197 PMC6617240

